# Value-Driven Pediatric Supracondylar Humerus Fracture Care: Implementing Evidence-Based Practices

**DOI:** 10.5435/JAAOSGlobal-D-24-00058

**Published:** 2024-03-28

**Authors:** Sonia Chaudhry

**Affiliations:** From the Department of Orthopaedic Surgery, Univeristy of Connecticut School of Medicine, Pediatric Orthopaedic and Hand Surgery, Connecticut Children's Medical Center, Hartford, CT.

## Abstract

Supracondylar humerus fractures are high-volume injuries in children; therefore, value-driven treatment has far-reaching implications for patients and families as well as healthcare systems. Children younger than 5 years can remodel posterior angulation. Most Type IIa fractures will maintain alignment after closed reduction. Many patients with surgical fractures can safely wait for nonemergent fixation. Outpatient surgery is associated with shorter surgical time, lower costs, and fewer return visits to the emergency department with no increase in adverse events. Type III fractures treated the following day do not have higher rates of open reduction, and patients with associated nerve injuries have no difference in recovery time compared with those treated more urgently. Pediatric-trained surgeons generally provide more efficient care (shorter surgical time and less after-hours surgery); however, their outcomes are equivalent to non-pediatric orthopaedic surgeons. Community hospitals have lower costs compared with teaching hospitals; therefore, transferring patients should be avoided when feasible. Postoperative care can be streamlined in uncomplicated cases to minimize radiographs, therapy referrals, and multiple visits. Splinting offers safer, lower cost immobilization over casting. With staffing shortages and an increasingly burdened healthcare system, it is imperative to maximize nonsurgical care, use outpatient facilities, and minimize postoperative requirements without negatively affecting patient outcomes.

Supracondylar humerus fractures (SCHFs) are the most common pediatric elbow injury, accounting for 15% of pediatric orthopaedic consults and 12% of pediatric orthopaedic surgeries.^1^ Optimizing value in this well-studied high-volume injury will, therefore, largely affect costs and patient experience. The ratio of healthcare benefits to costs defines value. To maximize value, we must decrease cost without negatively affecting patient outcomes or experience and improve benefits without commensurately increasing direct and indirect costs.

Decreasing defensive medicine, unnecessary transfers, after-hours surgery, and advanced and repeated imaging will all decrease costs. Usually, modalities that decrease cost concomitantly improve both patient experience and outcomes, for example, reducing opioids, office visits, and therapy. Operational issues such as chronic staffing shortages and lack of hospital beds during surges are also improved by maximizing nonsurgical care, limiting outpatient visits, and minimizing inpatient surgical care in lieu of outpatient surgery when possible. Longer term environmental concerns, such as operating room (OR) waste generation and car pollution from travel to and from visits, should also be factored into decision making.

Many patients and caregivers are not well equipped to make value-based decisions.^2^ Even physicians are left with many gray zones for best practices. For example, the 2012 American Academy of Orthopaedic Surgeons clinical practice guidelines are inconclusive regarding timing of treatment with concomitant neurovascular injury, pin removal, immobilization, and radiographs. This study will guide cost-effective and resource-saving practices while maintaining high quality of clinical outcomes based on the available evidence and author's clinical experience.

## Nonsurgical Care

### Prevention

The proverb ‘an ounce of prevention is worth a pound of cure’ comes to mind, as the best cost savings and decreased patient morbidity is fracture prevention. Vitamin D supplementation decreases the odds of fracture in children.^3^ Reduced sun exposure and obesity are risk factors for the potential need for supplementation.^4^ Just as the ‘Own the Bone’ campaign encouraged orthopaedic surgeons to play an active role in the bone health of elderly patients, families should be encouraged to improve the quality of children's diet, vitamin D intake, and activity levels while caring for pediatric fractures.

### Nondisplaced and Minimally Displaced Fractures

Nonsurgical treatment is indicated for Type I (nondisplaced, nonangulated) fractures. Comprehensive nonsurgical fracture care consists of immobilization, serial radiographs to assess for displacement until stabilization around 3 weeks, and then allowing early range of motion during final fracture healing (around 6 weeks). Multiple studies of Type I fractures have confirmed long arm splinting to be noninferior to casting for safe immobilization during fracture stabilization,^5,6^ and the author's general preference is a long arm plaster splint (Table 1). Serial radiographs can sometimes be skipped because the risk of displacement is low, and in very young children under 8 years, there is good remodeling potential. The third week visit is often the last because only a sling is used thereafter. With full understanding that the sling may end up mostly off the elbow, it still serves as a small reminder of caution to patients during the last 3 weeks of healing.

**Table 1:** Superiority of Splinting Over Casting

Factor	Splint (Long Arm Plaster)	Cast (Long Arm Fiberglass)
Fracture stability	Posterior slab can extend further proximal to the fracture on the humerus	Proximal extension limited by the axillary fold
Material cost	Plaster rolls $0.84 each	Fiberglass roll $1.97, plus stockinette
OR costs	No separate Current Procedural Terminology code	Separate CPT code
Application time	Plaster posterior and strut slabs prepared preoperatively, applied directly to Webril and covered with ACE wrap, can dry while patient waking up	Stockinette application, fiberglass application, stockinette folding, and more fiberglass application +/− drying and uni/bivalving time while under anesthesia
Room for swelling	Noncircumferential splint allows room to swell	Only possible with uni or bivalving, which requires more surgical time and risks cast saw injury in an anesthetized patient
Postoperative visits	None additional	Often 1 week visit recommended for overwrapping bivalved cast, incurring additional visit, material cost, and time
Removal	Able to be unwrapped by medical assistants, and is less scary than using a cast saw	Requires training in cast saw use, incurs risk of cast saw burns, and adds to patient anxiety
Medicolegal risk	If concerned for swelling and cannot be evaluated immediately, can be removed at home	Requires specialized equipment for safe removal and incurs risk of cast saw burns

Activity restrictions are empirically lifted 6 weeks after injury because low nonunion and reinjury rates preclude the need for radiographs at this time point. This streamlined approach minimizes missed school and work for families along with travel, and orthopaedic offices and radiology services are freed up for other patients. Most importantly, children can enjoy the mental and physical benefits of being physically active without waiting for full return to motion or the subjective quantification of radiographic callus.

### Nonsurgical Closed Reduction

Type II fractures (anterior cortical displacement > 2 mm with intact posterior hinge) are divided into IIa, with extension only, and IIb, which are additionally translated/rotated. Multiple studies of nonsurgical management of Type IIa fractures have shown over 70% rates of maintained closed reduction.^7,8^ Procedural sedation is associated with an 83% rate of successful nonsurgical management of Type IIa fractures compared with 56% in those without.^7^ One Level 1 trauma hospital found that their surgeons chose nonsurgical management in 75% of Type IIa fractures with a <5% need for surgical fixation.^9^ This was an exception to their overall 92% adherence to the 2014 American Academy of Orthopaedic Surgeons Appropriate Use Criteria (AUC), as the ‘appropriate’ recommendation for Type II fractures is surgical reduction with percutaneous pinning and reduction and casting is categorized as ‘may be appropriate,’ without distinction between IIa and IIb patterns.

While less than half of IIb fractures have a successful first reduction, and only 1/3 are able to complete nonsurgical care, those that do maintain successful alignment without surgery show no difference in clinical or radiographic outcomes compared with Type IIa fractures or compared with IIb fractures with surgical management.^10^ It is important to recognize that while surgery offers more consistent outcomes, in the setting of other factors that may increase the cost or morbidity of surgical management (distance, family resources, mobility, illness, lack of pediatric anesthesia), Type IIb fractures that are acceptably reduced and maintain their reduction at 1 week have similar outcomes to those treated surgically.^10^

**Figure 1:**
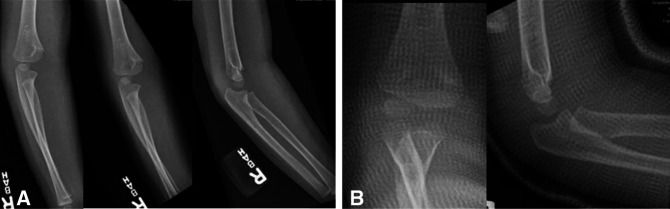
**A,** Radiographs showing the three views of an elbow fracture referred for surgical management because the capitellar ossification center appears angulated posterior to the anterior humeral line, that is, a Gartland Type II. **B,** Recognizing that this is not a true lateral, because the patient was likely unable to tolerate a lateral due to discomfort, the patient was immobilized for comfort, and radiographs repeated with the patient supine and the shoulder externally rotated, with the beam directed from medial to lateral. This provides the perfect lateral, with the ‘hourglass’ sign, and demonstrates this to be a more anatomic Gartland Type I, precluding the need for reduction or surgery.

The proximity of SCHFs to the elbow joint, desire to restore near-full range of motion, and relatively limited distal humeral remodeling potential make most surgeons intolerant of all but near-anatomic reduction. It is generally accepted that the ossification of the capitellum should be anterior to the anterior humeral line on lateral radiographs. The author emphasizes the need for perfect lateral radiographs to assess sagittal alignment and often will request repeat laterals obtained with the beam directed from medial to lateral through the elbow with the patient supine and externally rotating the shoulder for optimal alignment (Figure 1).

One study examining posterior malunions demonstrated complete or near-complete remodeling in 90% of their patients aged 2 to 8 years.^11^ It concluded that children younger than 5 years can remodel 100% displacement of their capitellum. This information should factor into decision making on the need for closed reduction, particularly if being considered with procedural sedation, which is associated with a higher rate of maintained alignment, but may be unnecessary if there is remodeling potential. In addition, as stated earlier, in children younger than 5 years with Type 1 fractures, weekly radiographs can be precluded because even in the event of angulation into extension, conversion to surgical management is unlikely.

## Surgical Care

### Location and Surgeon Training

Closed reduction and percutaneous pinning (CRPP) is currently indicated for most fully displaced fractures (Types IIb, III, and IV). Location, surgeon training, and timing of surgery have value implications. One teaching hospital charged an average of $6345 per CRPP.^12^ The cost breakdown in a descending order included the OR (67%), anesthesia (13%), fluoroscopy (11%), and implants (1%). One study comparing CRPP of mostly Type 2 fractures in a community hospital showed 44% cost savings over a teaching hospital, with over half of the savings coming from reduced OR costs and the remainder being from anesthesia, imaging, and supplies.^13^ Community hospitals are more likely to have non-pediatric orthopaedic specialists; however, outcomes are similar and satisfactory compared with pediatric fellowship-trained surgeons.^14^ The latter offers more efficient care, with pediatric surgeons having shorter surgical time (average 13 minutes), less fluoroscopic time, more delayed surgery over 12 hours, and fewer postoperative visits.^14,15^ One study also showed pediatric orthopaedic surgeons to have more optimal pin constructs and lower rates of iatrogenic nerve injuries (all of which resolved), but both groups ultimately had no differences in major loss of reduction.^16^ Another study found rates of open reduction, postoperative Baumann angle, and complications to be similar regardless of training background.^15^ Value is, therefore, not routinely improved by transferring patients to surgeons with pediatric training given the higher monetary and time costs of travel to further facilities for surgery and subsequent follow-ups. Despite the cost, however, transfers will continue to appropriate for certain situations to optimize patient care.

### Timing of Surgery

Timing of surgery also has value implications. SCHF surgeries performed between 11pm and 6am are associated with more malunions despite equivalent rates of open reduction, surgical time, and complications,^17^ although importantly these ‘overnight surgeries’ tend to be on more severe fractures. There has been a shift toward outpatient treatment, which reduces surgical time and costs.^18^ In New York, outpatient management went from 23% in 2009 to 59% in 2018, with both immediate and delayed cost savings. Inpatient surgeries billed $16,097 vs outpatient costs of $9752. Outpatients had fewer return visits to the emergency department within 1 month.^19^ However, outpatient care requires a caregiver at home, the absence of serious medical problems, and reasonable proximity to a surgical center. Although these are not modifiable factors, knowing these resource requirements can prompt surgeons to enlist the help of ancillary support as necessary to optimize value (eg, care coordination for transportation).

**Figure 2:**
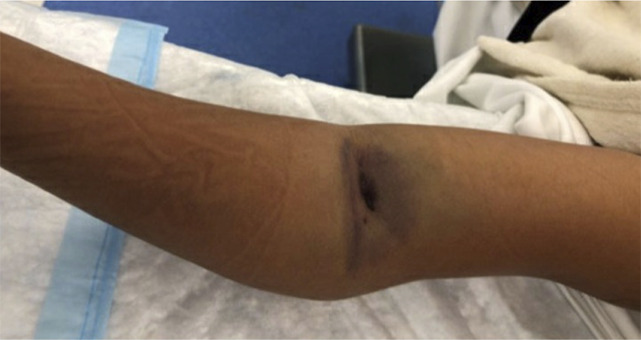
Image showing significant skin puckering and displacement seen with this fracture, and while it is ideal to provide gentle traction during timely initial evaluation and splinting, emergent after-hours surgery has not been shown to improve outcomes.

Treatment within 18 hours is tracked as a quality metric for the US News and World Report pediatric hospital ranking, despite multiple studies demonstrating the safety of delay in most situations. A large multicenter study of patients with anterior interosseous nerve palsies who underwent CRPP at an average of 15 hours (range 2 to 36) demonstrated no cases of compartment syndrome, and the authors concluded that a delay up to 24 hours did not increase time to nerve recovery or other complications.^20^ A single institution reported performing 65% of their CRPPs of Type III and IV SCHFs the following day with an average delay of 12 hours and found that timing, vascular status, nerve injury, and lateral displacement did not correlate with complications^21^ (Figure 2). Their complication rate only correlated with younger ages. The authors proposed early treatment for abnormal neurovascular status and next-day treatment for the remainder. Interestingly, they found that surgeons in practice for less than 15 years were 14 times more likely to treat early versus those in practice longer, with no difference in their complication rates.^21^ Another study looked at surgery within 24 hours for Type III fractures and found that those treated before or after 12 hours had no difference in surgical time, reduction quality, or rate of opening.^22^ It is important to keep in mind that while a delay in treatment is acceptable, a delay in evaluation is not benign because without assessment for open injuries, concomitant fractures, compromised perfusion, and compartment syndrome, fractures may be inappropriately triaged.

### Surgical Preparation

**Figure 3:**
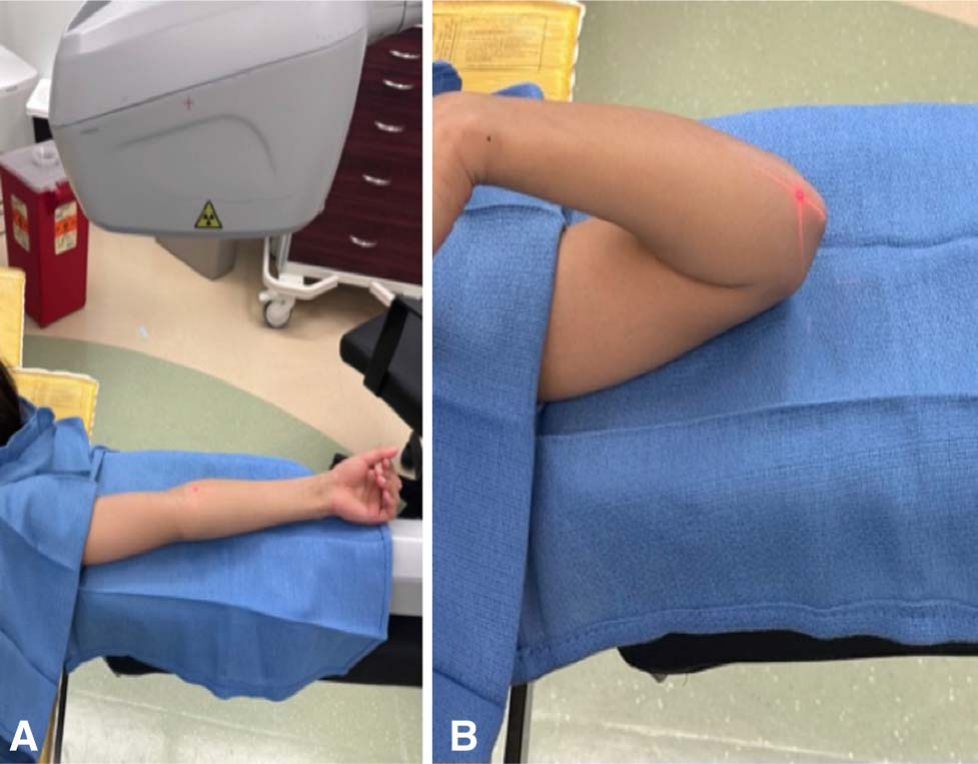
Images of the patient positioned supine with the arm abducted and extended onto a regular arm board. The mini-fluoroscopy is positioned atop the arm board with the laser localizer at the fracture line, which precludes the need for a radiolucent board or positioning the patient at the edge of the bed, which keeps their head safely positioned more centrally on the OR table. After prepping the arm atop the undraped fluoroscopy unit, two blue towels are placed longitudinally under the arm, and one towel is placed to cover the patient’s shoulder and face (**A**). When the elbow is flexed, the proximal towel keeps the hand from being contaminated (**B**).

**Figure 4:**
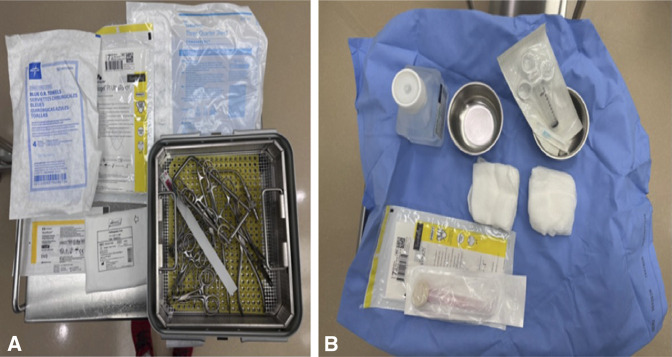
Photographs showing supplies for a minimal setup (**A**), specifically the three-quarter drape to cover the prep table, sterile blue towels, gloves, Xeroform, felt, and a ‘pin set,’ with the relevant instruments listed in Table 2. Prep bowls and gauze are also helpful so that saline can be used to clean the elbow before splinting, and a local anesthetic can be injected about the pin sites and fracture (**B**).

OR preparation can be optimized to minimize cost. It is the author's practice to avoid antibiotics in all CRPP cases. One study found an infection rate of 0.6% in 337 patients who received antibiotics and 0.4% in those who did not.^23^ Another series of infections after CRPP demonstrated 1/3 to be associated with a wet cast;^24^ therefore, effort should be directed at counseling families on the importance of cast care. Use of a semisterile technique using sterile gloves and towel draping, forgoing gowns and drapes, is safe, with no pin site infections in over 300 cases.^25^ This saves on setup time, environmental waste, and OR costs. The author has incorporated this by positioning an undraped mini-C-arm under the elbow at the start and prepping and towel draping the elbow atop the machine, which also precludes the cost and waste of the large plastic fluoroscopy drape (Figure 3). A small prep table containing a ‘pin set,’ or a few individually opened instruments, is set up, with additional instruments available but not open should the case become more complex (Figure 4). As preference card editing has been well established as one of the most effective ways to cut down on operating room monetary and environmental costs, Table 2 lists the instruments and supplies to be open for a typical CRPP case.

**Table 2:** Preference Card Sample for a Supracondylar Humerus Fracture Closed Reduction And Percutaneous Pinning Minimalist Setup

Instruments	Supplies
Prep bowls (for saline, local, etc.)	Three-quarter sheet (to drape prep table)
Power driver and pin collets	Saline
Wires (smooth)	Blue towel pack
Wire cutter	Sterile gloves
Needle driver or plier	Prep stick
	Xeroform/felt
	Gauze
	Syringe/needle (for local anesthetic injection)
	Sterile Webril

### Surgical Technique

**Figure 5:**
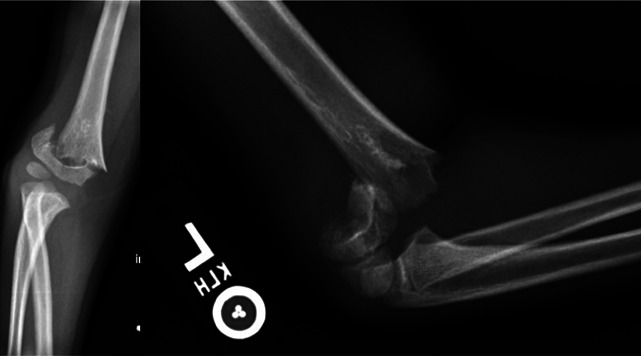
Anteroposterior and lateral radiographs of a Gartland Type III fracture with posterolateral displacement and rotation is demonstrated. Supination and traction will improve axial and coronal alignment, respectively, before flexing the fracture to restore the sagittal plane.

The surgical technique varies, yet literature does exist to guide various aspects. Closed reduction should be performed whenever possible, with validated reduction maneuvers being pronation for posterior and posteromedial displacement and supination for posterolateral displacement (Figure 5). Risks of requiring open reduction include flexion types (IV) and coronal displacement >7 mm,^26^ so while these fractures are still predominantly treated with CRPP, the potential need for opening could be anticipated and factored into a more standard prep-and-drape setup. Loose bone fragments on x-ray are seen in 2.6% of patients and are not an indication for opening because these fragments absorb or reunite eventually.^27^

**Figure 6:**
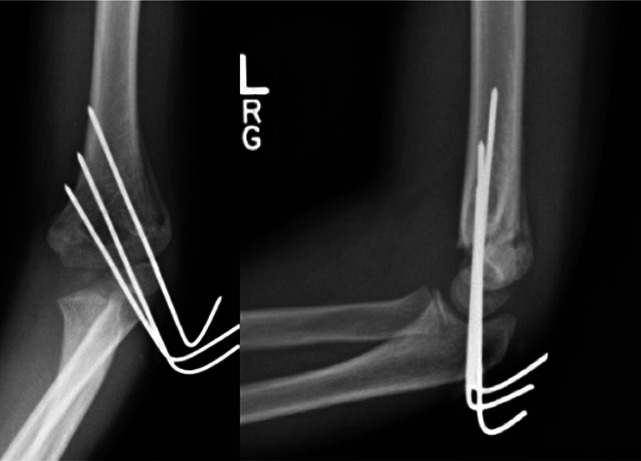
Three lateral entry divergent pins stabilized this Type III fracture for 3 weeks. Purchase in the olecranon fossa provided additional fixation compared with bicortical purchase alone. With the callus now present, there is enough stability such that pins can be removed in clinic because they were cut and bent to rest outside the skin percutaneously.

Divergent retrograde oblique pins, at least two for Type II injuries and three for Types III and IV, are ideally placed from the lateral side (Figure 6). Medial pinning carries a 4% risk of iatrogenic ulnar nerve palsy, and although 90% of palsies recover,^28^ this is stressful for both families and physicians and can increase the number of visits. The author avoids medial entry pins except when medial column comminution precludes adequate bicortical purchase for all the lateral entry pins. On these occasions, at least two lateral entry pins should still be used to stabilize the reduction in flexion, and then the elbow can be extended for medial pin entry. In cases where wires skive off the second cortex and travel up the humeral shaft, a larger caliber lateral wire is used instead of switching to a medial pin. Pins should be bent and cut to rest outside the skin and wrapped with Xeroform to minimize pin-skin interface motion. Buried pins back out less but are associated with more skin irritation, outpatient visits, costs, and burdens.^29^

### Imaging

Intraoperative and postoperative imaging should be optimized to reduce cost and radiation exposure. One study found that fluoroscopy time and radiation dose were not affected by resident participation, instead it varied based on the fracture type, number of pins, and operating surgeon.^30^ For this reason, although the author provides hands-on teaching to trainees, the mini-C-arm pedal is always controlled by the attending surgeon to minimize exposures to only those that are necessary to guide safe pin placement and ensuring the elbow is held steady and centered with laser guidance to avoid blurry or suboptimal shots. In addition, the author makes a point to save views that demonstrates the maximum residual displacement and angulation. Some amount of nonanatomic reduction is acknowledged to be present and acceptable, and any subsequent postoperative radiographs, for example, after a fall or report of new pain, can be truly assessed for a change in alignment warranting a return to the OR for revision versus a different view but unchanged alignment from index surgery.

Mini-fluoroscopy offers several advantages over large-C-arms because staff over 3 feet away do not require lead shielding, a fluoroscopy technician is not needed during the case, and pulsed dose settings minimize exposure to both patients and surgeons. Additional intraoperative fluoroscopy after pinning and immobilizing does not change management.^31^ Finally, either pre or post-pin removal radiograph can be ordered, but doing both does not add value or change management.

### Pain Management

Pain management plays an important role in patient experience and can influence the number of phone calls and unexpected follow-ups to the surgeon's office. Intraoperative ketorolac reduces pain and inpatient opioid use and length of stay by 50%, along with lower inpatient hospitalization, resulting in a 34-fold return-on-investment cost savings, with no difference in complications.^32^ Postoperative acetaminophen is as effective as narcotics after CRPP, but with fewer adverse effects.^33^ Pain should be discussed postoperatively, both to set reasonable expectations and to know what is concerning for pain out of proportion, which could indicate compartment syndrome. Strategies to minimize opioid use include preoperative informed consent for opioids^34^ and standard order sets.^35^ The author's practice is to avoid prescribing opioids, using intraoperative bupivacaine injection about the fracture and pin sites along with ketorolac administration, in conjunction with education about combining acetaminophen and ibuprofen for maximum nonopioid analgesia. Elevation above the level of the heart and active finger movement to pump edema back can mitigate postoperative swelling and pain, which is why the author avoids routine sling use, which discourages both elevation and finger movement.

### Immobilization

Another postoperative factor that can play a large role in improving value is the type of immobilization. The author has a strong preference for plaster splinting over fiberglass casting for both nonsurgical and postoperative immobilization (see Table 1) because a splint allows for more proximal immobilization up the posterior humerus while the proximal extent of a cast is limited by the axillary fold. Plaster splinting also decreases direct costs with lower material costs and less OR time. Plaster slabs can be prepared ahead of time while fiberglass needs stockinette application, layered wrapping, folding of stockinette edges, additional wrapping, and then drying time before valving. Experts recommend a curing time of 10 minutes for fiberglass and 12 minutes for plaster^36^ before cast saw use, and still the risk of burns is higher than baseline because patients are under anesthesia. Patients with valved casts often have a separate visit for fiberglass overwrapping and later are subjected to a loud/scary cast saw for removal. The use of postoperative splinting over casting improves the flow of clinic as cast beds are not necessarily needed, and staff without cast saw knowledge can unwrap the splint.

Multiple studies have shown no difference in unscheduled returns or secondary displacement with splinting versus casting.^37,38^ The author has had good success with ∼10 layers of plaster for a posterior splint supplemented with a ∼5-layered lateral slab to cover pins, with an ACE wrap applied directly onto the plaster while drying to make it harder to remove.

### Postoperative Care

Postoperative care should ensure appropriate clinical recovery and radiographic healing with the least burden to patients, caregivers, healthcare facilities, and the entities paying for the incurred costs. The author has previously published an algorithm for streamlining postoperative care.^39^ Routine fractures receive only one postoperative visit around 3 to 4 weeks for a 2-view radiograph, pin removal, instruction on a home exercise program, three additional weeks of activity restriction and sling use, and counseling on the expected course and when/why to contact the office for additional visits. Because patients have high anxiety regarding pin removal, this is performed at the end of the visit so that children can then leave the clinic and recover in a more comfortable environment. This is why we radiograph children out of their splint before pin removal, and no post-pin removal radiographs are repeated because they do not change management. We avoid visits for motion checks because families can assess for this themselves or demonstrate their motion through telemedicine. Therapy referral is not routinely used because worse 9 and 15-week post-injury scores and higher 9-week anxiety scores have been demonstrated with therapy.^40^ Patients with a home exercise program have similar return time to activities of daily living and sports with no difference in elbow motion compared with those who do therapy. If families specifically request therapy, they are counseled on these data, but we also encourage families to contact the office for a referral if motion is not near-normal by 4 months post injury.

## Summary

This article seeks to provide evidence-based recommendations for improving patient outcomes and experience while decreasing healthcare costs associated with pediatric SCHFs. Fracture prevention is ideal, which is why orthopaedic surgeons should appropriately counsel patients on vitamin D intake and the importance of having healthy body weight. Nonsurgical care should be used in nondisplaced and successfully closed reduced SCHFs. While routine management consists of immobilization and weekly radiographs for 3 weeks, with conversion to surgical management for loss of reduction, fractures with sagittal plane remodeling potential, particularly in children younger than 5 years, can forgo some of this for efficiency.

For surgical management, the cost of routine transfer to pediatric fellowship-trained surgeons and specialty hospitals is not justified given the similar outcomes. Outpatient surgery offers cost savings over inpatient management. Emergent after-hours surgery in the setting of Type III and IV injuries, even in the setting of nerve injury or diminished pulse, is not required as long as the hand is well perfused and closely monitored.

It is safe and economical to forgo preoperative antibiotics, gowns, and full drapes. Using a semisterile technique, surgeon-operated mini-C-arm, and lateral pins when possible will improve surgical efficiency. Subcutaneous local, intravenous ketorolac, and oral acetaminophen and ibuprofen should preclude the need for narcotics. A plaster long arm splint provides strong protection at a lower cost and higher safety profile compared with a fiberglass long arm cast. Routine postoperative care can be streamlined to a single visit and radiograph for pin removal.
